# An observational laboratory study to evaluate an anesthetic gas adsorber without anesthetic gas scavenging system

**DOI:** 10.1186/s12871-025-03223-7

**Published:** 2025-07-30

**Authors:** Katja Nickel, Christian Thoben, Christiane E. Beck, Jan Karsten, Terence Krauß, Alexander Nitschke, Moritz Hitzemann, Stefan Zimmermann, Sebastian Heiderich

**Affiliations:** 1 Clinic of Anesthesiology and Intensive Care Medicine, Hannover Medical School, Carl-Neuberg-Str. 1, 30625 Hannover, Germany; 2 Institute of Electrical Engineering and Measurement Technology, Department of Sensors and Measurement Technology, Leibniz University Hannover, Hannover, Germany

**Keywords:** Anesthetic gas adsorber, Sevoflurane, Volatile anesthetics, Global warming potentials, Ion mobility spectrometry, Anesthetic gas scavenging system, Fresh gas flow

## Abstract

**Background:**

Volatile anesthetics are known to be potent greenhouse gases and a significant source of per-and polyfluoroalkyl substances (PFAS) “forever chemical” pollution. The latter is arguably an additional strong reason to stop the emission of anesthetic exhaust gases into the atmosphere on its own. In clinical practice large proportions of used volatile anesthetics are released into the environment via anesthetic gas scavenging systems. Anesthetic gas adsorbers have been developed to bind volatile anesthetics for later extraction and reusage. They may have the potential to replace anesthetic gas scavenging systems which would have a beneficial effect on the energy consumption of hospitals. However, studies are needed to ensure effective elimination of volatile anesthetics via anesthetic gas adsorbers without gas scavenging systems.

**Objective:**

To evaluate an anesthetic gas adsorber during simulated ventilation.

**Design:**

A bench study.

**Setting:**

An anesthesia machine was connected to an anesthetic gas adsorber (CONTRAfluran™ system, Zeosys Medical, Luckenwalde, Germany) without the use of an anesthetic gas scavenging system. A test lung was ventilated with sevoflurane and oxygen. Sevoflurane concentrations in parts per million (ppm) were detected directly from the anesthetic gas adsorber exhaust outlet using ion mobility spectrometry with gas chromatographic preseparation. A total of 6 experiments were conducted with alternating fresh gas flows, sevoflurane concentrations, humidified air and CO_2_ insufflation.

**Main outcome measures:**

Absolute sevoflurane concentration.

**Results:**

Sevoflurane concentration remained < 1 ppm as long as the canisters were not saturated. At higher fresh gas flow, the breakthrough time of the anesthetic gas adsorber decreased proportionately. Humidified air and CO_2_ insufflation had only a minor influence on the breakthrough time.

**Conclusion:**

The anesthetic gas adsorber did not leak relevant sevoflurane concentrations through the exhaust outlet when used without an anesthetic gas scavenging system. When the canister came close to saturation, the post adsorber exhaust sevoflurane concentration progressively increased–indicated by first the yellow-light and subsequently the red-light warning of the anesthetic gas adsorber system. Continuation of the ventilation with a fully saturated canister resulted in ambient room contamination of up to 12.4 ppm sevoflurane, which though undesirable is still low when compared to mask induction.

## Key points

• Volatile anesthetics are potent greenhouse gases and are released into the atmosphere via anesthetic gas scavenging systems.

• Anesthetic gas adsorbers have been developed to capture volatile anesthetics using activated carbon.

• This study evaluates if an anesthetic gas adsorber without anesthetic gas scavenging system leaks relevant sevoflurane concentrations during simulated anesthesia.

• Sevoflurane concentrations measured directly at the exhaust outlet remained < 1 ppm as long as the adsorber canisters were not saturated.

• Breakthrough time was decreased with higher fresh gas flow, while humidity and CO_2_ had only minor influence.

## Introduction

The healthcare sector in industrialized nations accounts for a surprisingly high proportion of greenhouse gas emissions (4.4%) [1]. To assess the carbon footprint of individual products, life cycle assessments are carried out, for which three scops are defined for accounting and reporting. Scope 1 comprises all direct emissions within the organization’s boundaries, Scope 2 the indirect emissions resulting from electricity consumption and Scope 3 the remaining indirect emissions that arise as a result of the organization’s activities [2]. Greenhouse gases also include volatile anesthetics, of which only a small proportion are metabolized (desflurane 0.02% [3], sevoflurane 5% [4], isoflurane 0.2% [5]). Therefore, a large proportion is discharged directly into the atmosphere via anesthetic gas scavenging systems (AGSS). Volatile anesthetics and some of their degradation products, such as hexafluoroisopropranol (HFIP) and trifluoroacetic acid (TFA), are classified as per- and polyfluoroalkyl substances due to the one fully fluorinated methyl group. These substances are also referred to as ‘forever chemicals’, because they can remain in the environment for a long time [6]. This is yet another good reason not to release the exhaust gases into the atmosphere.

In recent years, anesthetic gas adsorbers have been developed that collect the gas, e.g. CONTRAfluran™ (Zeosys, Luckenwalde, Germany), Deltasorb^®^ (Blue-Zone Technologies, Ontario, Canada) or SID-Dock/SID-Canisters^®^ (SageTech Medical, Paignton, UK). They prevent the gas from being released into the environment and allow a proportion of it to be recovered, although this is less than 50% [5, 7, 8]. The extent of the impact of anesthetic gases on global warming is currently being discussed by climate researchers. Global warming potentials (GWP) serve as a tool for comparing the effect of individual greenhouse gases on global warming [9]. In recent years, there has been an extensive and complex debate in the climate literature about the relevance of simple emission indicators, such as the GWP, for short-lived climate pollutants [10–12]. Emissions of long-lived (and therefore cumulative) gases have a fundamentally different impact on the planetary energy balance compared to short-lived climate pollutants. Due to the selective release of anesthetic gases into the atmosphere, their short lifespan and thus low accumulation in the atmosphere, anesthetic gases may not have a major impact on global warming. Nevertheless, the impact and GWP over a shorter period (20 years) is considerably higher (sevoflurane GWP_20_ = 702, desflurane GWP_20_ = 7020, isoflurane GWP_20_ = 1930) [13]. 

However, Slingo and Slingo assume that the production, distribution and transport of the filters for the distillation of volatile anesthetics are more likely to lead to additional CO_2_ emissions that could be relevant for global warming [14]. The significant greenhouse gas emissions savings also help to avoid PFAS emissions by reducing releases to the environment and lowering production rates. A lifecycle assessment (LCA) of the individual anesthesia procedures with anesthetic gas adsorbers could provide a statement about the CO_2_ footprint. There is an LCA by Hu et al. for anesthetic gas adsorbers, though not all aspects were examined [15]. Another advantage of anesthetic gas adsorbers could be that AGSS consume a lot of energy via compressed medical air to drive ejectors and would no longer be necessary [16, 17]. However, the anesthetic gas adsorber must be effective to prevent detectable leakage of volatile anesthetics. In this study we evaluate an anesthetic gas adsorber using an ion mobility spectrometer combined with gas chromatographic preseparation as analytical detector, capable of detecting sevoflurane in the ppb range.

## Methods

### Anesthetic gas adsorber system

An anesthesia machine (Primus^®^, Dräger Medical Deutschland, Lübeck, Germany) was disconnected from the AGSS and connected instead to an anesthetic gas adsorber (CONTRAfluran™ system, Zeosys Medical, Luckenwalde, Germany). This anesthetic gas adsorber consists of a plastic canister (polypropylene) filled with activated carbon made from coconut shells. Due to the coarse-pored structure, the exhaled air from the anesthetic workstation flows passively through the anesthetic gas adsorber and exhausts into the room air. Volatile anesthetics are physically bound in the process. Once the adsorber is almost saturated and the output of volatile anesthetics into the room exceeds 1200 ppm, a yellow-light warning appears on the anesthetic gas adsorber fill level control unit (SENSOfluran™, Zeosys Medical, Luckenwalde, Germany), followed by two yellow lights when exceeding 1500 ppm. When the adsorber is full (exceeding an output of 2000 ppm volatile anesthetic) a red-light and an acoustic warning informs the anesthesia provider to change out the adsorber canister. The sensor utilised in the SENSOfluran™ is a thick film metal oxide semiconductor sensor which changes its resistance depending on the substance and concentration present within the sensor, including sevoflurane. Later, the anesthetic gases can be retrieved from the activated charcoal canister in an automated factory process [18]. 

### Simulated anesthesia experiments

A test lung (SelfTestLung™ Dräger Medical Deutschland, Lübeck, Germany) was connected to the anesthetic machine and ventilated with 100% oxygen and different concentrations of sevoflurane at different fresh gas flows. A volume-controlled ventilation was chosen with a respiratory rate of 12⋅min^−1^, a positive end expiratory pressure of 5 mbar and a tidal volume of 500 ml. At a 120-second interval, air samples were taken directly from the anesthetic gas adsorber exhaust outlet. Any sevoflurane exiting the exhaust of the adsorber was then detected using ion mobility spectrometry with gas chromatographic preseparation (GC-IMS) and compared to the indicator warning light of the anesthetic gas adsorber. For every experiment, a new anesthetic gas adsorber canister was used. A total of 6 experiments were conducted:

Experiment 1 was a stress test in which the fresh gas flow (FGF) rate was set to 18 L⋅min^−1^ and the vaporiser set to 8 vol% sevoflurane to produce the highest sevoflurane exposure as possible [19]. Once the red-light warning appeared, the FGF was reduced to 2 L⋅min^−1^. The aim of this experiment was to create a worst-case scenario with a clinically unrealistic excess exposure of sevoflurane to the adsorber to evaluate whether the anesthetic gas adsorber is able to adsorb high concentrations of sevoflurane presented at a high FGF, and if a saturated adsorber canister then leads to clinically relevant contamination of the room air with sevoflurane.

Experiments 2–4 were designed to assess a possible correlation between FGF and breakthrough time of the adsorber canister (defined as time until red-light warning light appears). The FGF was set to 1 L⋅min^−1^ (experiment 2), 2 L⋅min^−1^ (experiment 3) and 4 L⋅min^−1^ (experiment 4). The Vaporiser was set to 2 vol% sevoflurane.

In experiments 5 and 6 a patient’s metabolism were simulated to evaluate if humidity or CO_2_ have an impact on the breakthrough time. In experiment 5 the inspiratory O_2_-sevoflurane mixture was humidified with an F&P 950™ humidifier (Fisher & Paykel Healthcare, Auckland, New Zealand). The humidifier was set to an estimated dew point of 31 ± 2°C, at which a humidification performance of > 12 mg·L^−1^ is guaranteed by the manufacturer. In experiment 6, additional CO_2_ was insufflated directly into the test lung at a rate of 1 L⋅min^−1^ to simulate a patient with a high metabolism. The exhaled CO_2_ was bound with a soda-lime absorber (Infinity ID CLIC Absorber 800+, Dräger Medical Deutschland, Lübeck, Germany) with 78–84% Ca(OH)_2_, 2–4% NaOH, 14–18% H_2_O and < 0.1% ethyl violet (Table 1). Details of the experiment protocols are summarised in Table 1.

**Table 1:** Protocol of experiments 1-6. FGF, fresh gas flow; *FGF was reduced to 2 L×min^-1^ once red-light warning appeared

Experiment no.	FGF (L×min^-1^)	Vaporiser setting (Vol%)	Humidifier	CO_2_ (L×min^-1^)
1	18*	8	-	-
2	1	2	-	-
3	2	2	-	-
4	4	2	-	-
5	4	2	on	-
6	4	2	on	1

### Calculation of Sevoflurane consumption

The following equation can be used to estimate the consumption or storage capacity of the simulated anesthesia experiments. It should be noted that the molar volume of a gas $\:{V}_{\text{m}}$ and the fresh gas flow rate refer to standard conditions (20°C and 1013.25 hPa).

$$\:m=\frac{\text{v}\text{o}\text{l}\%}{100}\cdot\:\frac M{V_\text{m}}\cdot\:FGF\cdot\:t$$

In this case, the molar volume is 24.055 L mol^−1^ and the molar mass of sevoflurane is 200.056 g mol^−1^. The mass of sevoflurane adsorbed can be estimated using the measured volume fraction of sevoflurane (vol%) from the anesthetic machine, the set FGF rate and the corresponding time (t). It should be noted that, according to the manufacturer’s specifications, the FGF can have an error of ± 10%, therefore the calculation of the mass here can only ever be an estimate.

### Ion mobility spectrometry

A compact, high-resolution ion mobility spectrometer (IMS) with gas chromatographic (GC) preseparation was utilized to ascertain the sevoflurane concentration. A detailed description of the system can be found elsewhere [20]. An IMS separates different substances on basis of their ion mobility, which is determined by the drift velocity of their respective ions through a drift gas under the influence of an electric field. The used IMS achieves a mobility resolving power of *R* = 70 (drift time/full width at half maximum). The IMS is driven by self-developed electronics for the drift voltages, the ion gate control, and the data acquisition, as presented by Hitzemann et al. [21] In contrast to previous publications, only the negative polarity is employed in this study for the IMS. A 20 m Restek RTX volatiles GC column with an inner diameter of *I*_*D*_ = 530 μm and stationary phase layer thickness of *d*_*f*_ = 2 μm is used isothermally at 40°C for preseparation. The sample is injected into the carrier gas stream via a defined sample loop volume of 250 µl, which is directed onto the GC column. Following the separation of the sample over time in the GC, the sample components flow with the carrier gas into the IMS ionization area, where the sample analytes are ionized and detected by the IMS. Consequently, all peaks observed in GC-IMS measurements are characterized by their ion mobility, GC retention time, and peak area, which is related to the concentration of the compound. The detection limit of the present method is 70 ppb. The measurement error is 10% of the measured value. However, for concentrations in the upper measurement range the measurement error increases to 20%.

## Results

**Fig. 1:**
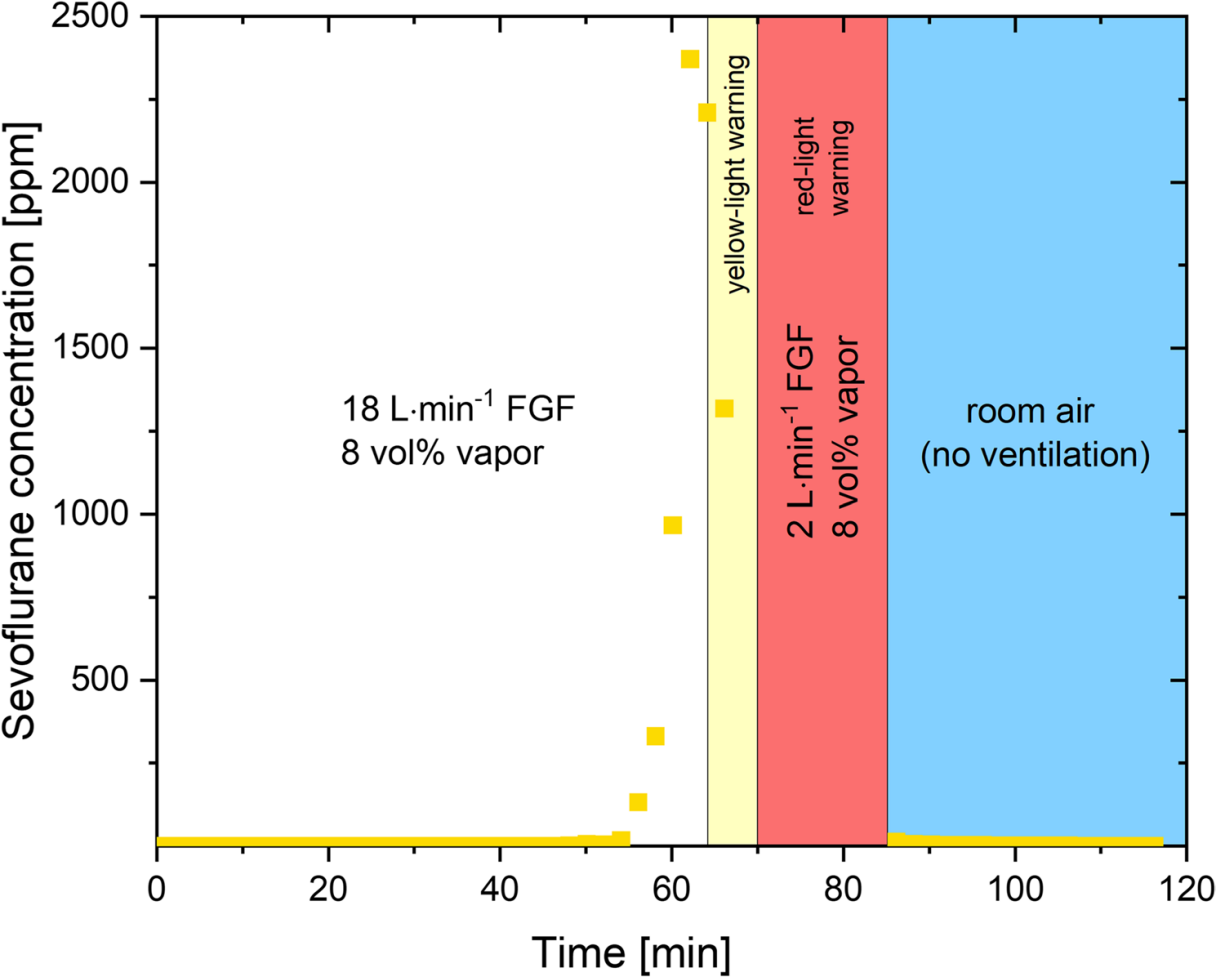
Sevoflurane concentrations measured directly downstream of the anesthetic exhaust adsorber during 18 L×min^-1^FGF and 8% vapor ventilation. FGF was reduced to 2 L×min^-1^once red-light warning appeared. Following ambient room air measurement at 1 m from the anesthetic exhaust of the adsorber. FGF, fresh gas flow

**Fig. 2:**
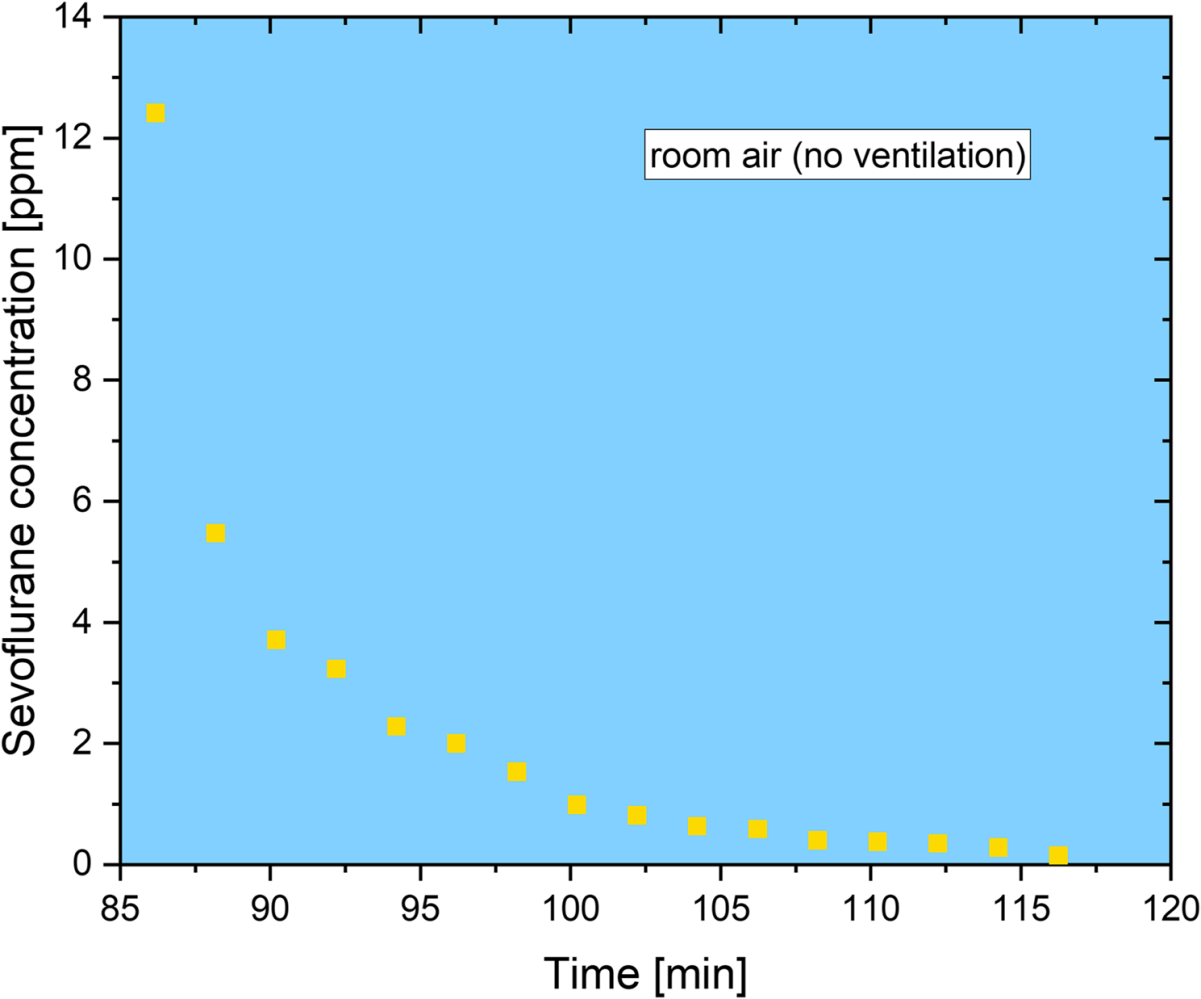
Zoom into the room air measurement with the sevoflurane concentrations over time after experiment 1, incl. 15-minute ventilation with red-light warning, at 1 m from the anesthetic exhaust of the adsorber

**Fig. 3:**
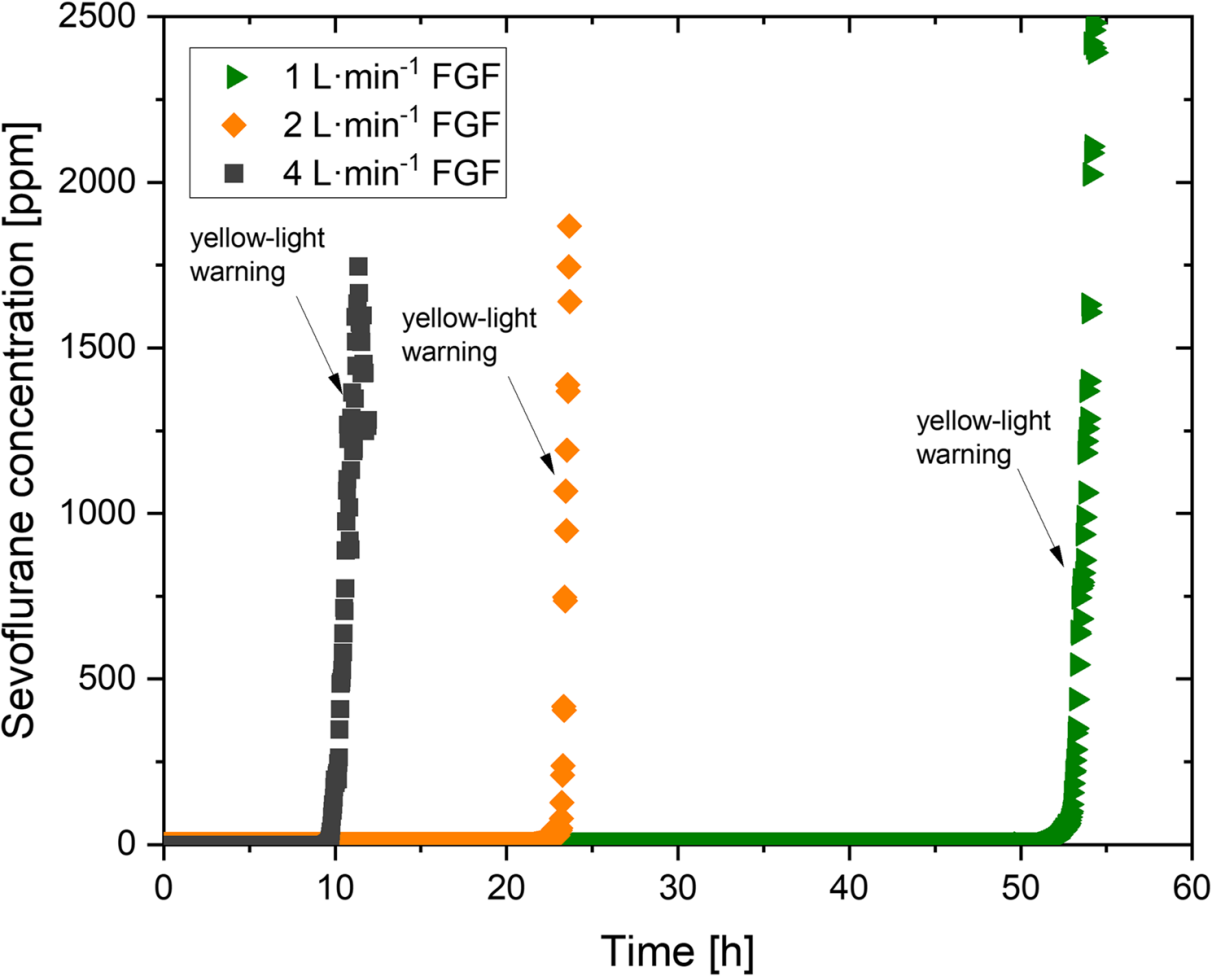
Sevoflurane concentrations over time for different FGF (1 L⋅min^−1^ green, 2 L⋅min^−1^ orange, 4 L⋅min^−1^ dark gray) measured at the anesthesia exhaust of the adsorber. Arrows indicating the time of first yellow-light warning. FGF, fresh gas flow

The experiments of this study were conducted from January until March 2024. In the initial phase of the stress test (experiment 1), the FGF was set to 18 L⋅min^−1^, with the sevoflurane vaporizer set to 8%. The inspiratory sevoflurane concentration was 5.9–3.7 vol% depending on the vapor level. This configuration was maintained until the initial yellow-light warning indicator was triggered (64 min). The yellow-light warning was displayed for a period of six minutes until the sensor directly issued the final red-light warning (70 min). Thereupon, the FGF was reduced to 2 L⋅min^−1^ to prevent further concentration increases. Nevertheless, adsorber sevoflurane exhaust concentrations continued to rise, and the sensor continued to issue the red-light warning for the 15-minute duration of the test section. A room air measurement was conducted immediately following the completion of the test, at a distance of 1 m from the anesthetic exhaust of the adsorber. The room was a 53.7 m^3^ operating theatre side room with an exchange rate of 4.8 vol⋅h^−1^ without recirculation. The initial measured concentration of 12.4 ppm dropped to below 5 ppm within four minutes (Fig. 1). A detailed representation of the measurement can be seen in Fig. 2. The mean sevoflurane concentration observed over the subsequent 30 min was 2.2 ± 3.1 ppm. Figure 3 illustrates a comparable curve progression between the FGF and the duration of anesthetic adsorber utilization. As the FGF value increases, the breakthrough time of the anesthetic exhaust adsorber decreases. Furthermore, the dependence on the FGF is evident in the gradient of the change in concentration behind the anesthetic exhaust adsorber over time, which increases with the FGF. This is also reflected in the time intervals between the initial yellow-light and the subsequent red-light warning. With a FGF of 1 L⋅min^−1^, a period of 226 min elapses between the warnings. At a FGF of 2 L⋅min^−1^, this period is reduced to 70 min, while at a FGF of 4 L⋅min^−1^, it is further reduced to 63 min. A mathematical estimate indicates that reducing the FGF from 4 L⋅min^−1^ to 1 L⋅min^−1^ can result in an 24.5% increase in absorption capacity. It should be noted that this is the combined absorption capacity of the surfaces within the anesthesia machine (e.g. breathing tubes), the absorber lime and the anesthetic gas adsorber. The combined absorption capacity is a clinically relevant factor for the decontamination process of the anesthesia device for patients susceptible to malignant hyperthermia [22].

Additionally, low increasing concentrations of sevoflurane in the ppb range were detected. This value exceeded 1 ppm after 3080 min for a FGF of 1 L⋅min^−1^, after 1298 min at a FGF of 2 L⋅min^−1^, and the shortest time after 574 min for 4 L⋅min^−1^ FGF before the initial yellow-light warning of the SENSOfluran™ level indicator appeared. At the time of the initial yellow-light warning, the concentrations at the anesthetic gas adsorber exhaust outlet were 746 ppm, 1067 ppm, and 1365 ppm, respectively. The corresponding data for all experiments are summarised in Table 2.

**Table 2:** Results of experiments 1-6. Sevoflurane concentrations are measured at the anesthetic gas adsorber outlet and therefore are not equal to workplace contamination; time to red-light warning is defined as “breakthrough time”; FGF, fresh gas flow; * threshold value 1 ppm

Experiment no.	Time to first detected sevoflurane* (min)	Time to yellow-light warning (min)	Sevoflurane concentration at yellow-light warning (ppm)	Time to red-light warning (min)	Yellow-light to red-light warning interval (min)	Calculated sevoflurane mass until red-light warning (g)
1	46	64	2210	70	6	499
2	3080	3204	746	3430	226	685
3	1298	1408	1067	1478	70	563
4	574	660	1365	723	63	550
5	616	662	1294	700	38	547
6	580	640	710	680	40	517

**Fig. 4:**
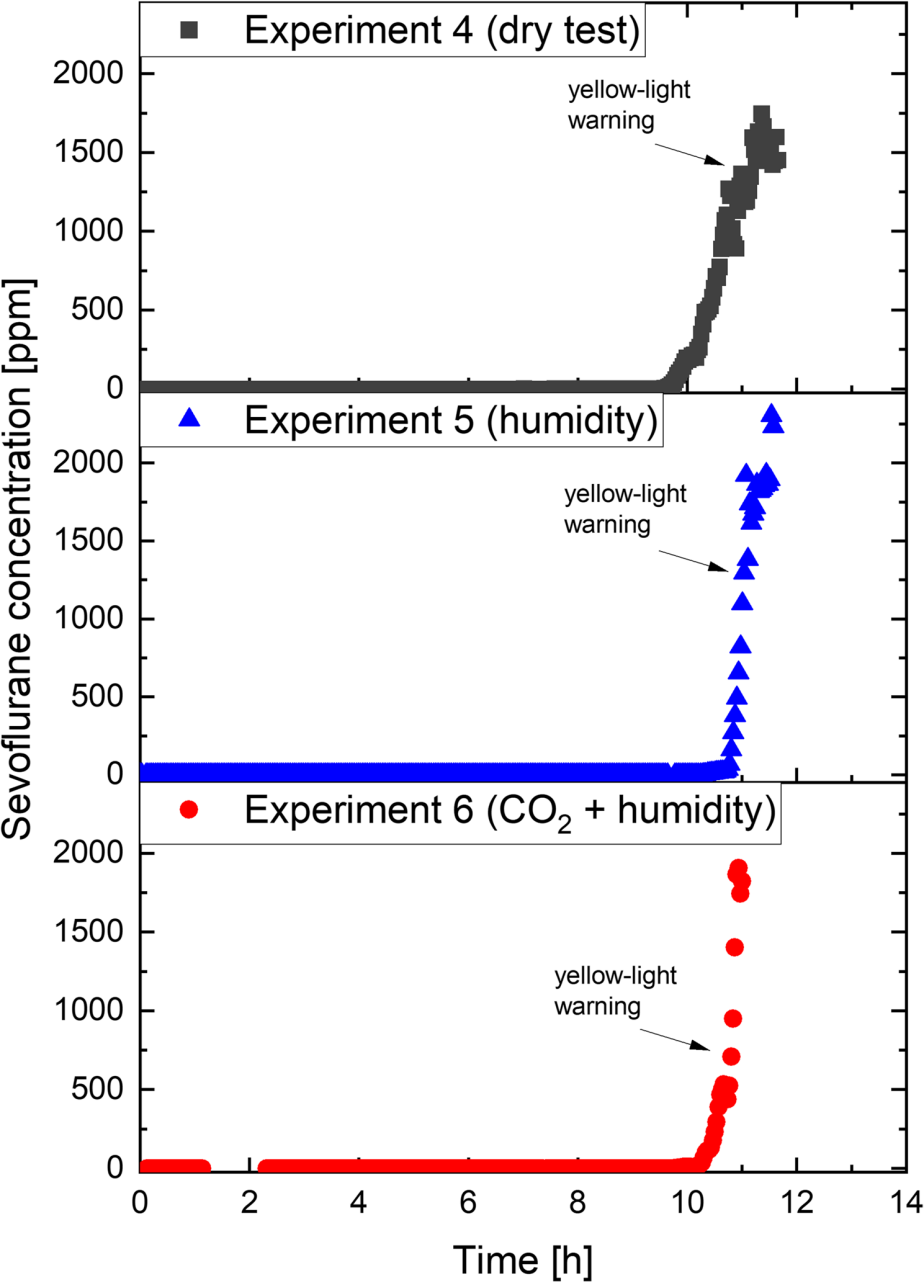
Sevoflurane concentrations over time for dry conditions (top, dark gray), with humidity (middle, blue), and insufflated CO_2_ plus humidity to simulate patient metabolism (bottom, red) measured at the anesthetic exhaust of the adsorber. Arrows indicating the time first yellow-light warning

In order to simulate the influence of the patient’s metabolism, the expiratory air was modelled more precisely with the aid of a humidifier (experiment 5 and 6) and by insufflating 1 L⋅min^−1^ CO_2_ (experiment 6). The FGF was set to 4 L⋅min^−1^ in these two experiments to compare the results with experiment 4. The corresponding sevoflurane concentrations over time are shown in Fig. 4. The simulated metabolism of the patient had only a minor influence on the breakthrough time of the anesthetic gas adsorber, with a difference of only 20 min between the experiments 5 and 6. However, it is noteworthy that the time between the first yellow-light warning and the red-light warning decreased to 38 min with humidification and 40 min with additional CO_2_ compared to 63 min with dry air.

## Discussion

In this study sevoflurane concentration (measured directly at the exhaust outlet of the anesthetic gas adsorber canister) remained undetectable down to the ppb range as long as the canister was not saturated. Even during the stress test (experiment 1) where clinically unrealistic concentrations of sevoflurane and a maximal FGF was chosen. Humidity and exhaled CO_2_ did not alter this result, only the absorption capacity was minimally influenced by CO_2_. However, as soon as the canister came close to saturation, a rising sevoflurane concentration was detectable, which is correctly indicated by the SENSOfluran™ fill level control unit (yellow-light and subsequently red-light warning).

Even after 15 min of operation with a red-light warning the ambient room contamination of sevoflurane (workplace contamination) only reached a lower ppm range of a maximum of 12.4 ppm. Therefore, it can be inferred that operation with the anesthetic gas adsorber does not result in relevant contamination of the surrounding environment. It is noteworthy that the tests were conducted in a relatively confined space within the operating theatre wing. Nevertheless, the concentration levels remained within the range observed in previous studies, as evidenced by a recent review of volatile anesthetic exposures in hospitals [23]. It is important to consider when using anesthetic gas adsorber systems that the room air sevoflurane concentration is affected by the room volume and the fresh air exchange rate without recirculation, and thus may vary in different rooms due to these factors.

With the GC-IMS three unknown substances were detected, which could be potential reaction products of sevoflurane [24–26]. The exponential relationship between breakthrough times and FGF may be attributable to the dynamics of the adsorption process and the effects of flow rate on contact time in the adsorber. At higher FGF, the gas has less contact time and or some channelling occurs with the adsorption medium in the anesthetic gas adsorber. This can lead to a reduction in the efficiency of the adsorption process. However, in this study, even at 18 L⋅min^−1^ FGF the sevoflurane concentration at the adsorber gas outlet remained < 1 ppm until the adsorber was saturated. At lower FGF, the contact time increases, which can lead to a higher adsorption capacity. Additionally, the filter design and the structure of the adsorption medium can influence the flow dependence of the adsorption process. If the adsorption is diffusion-limited, sevoflurane has more time to reach the activated carbon by diffusion at lower FGF [27]. 

### Limitations

It should be noted that a GC-IMS is not designed to measure concentrations as high as the sevoflurane peaks shown in this study. Consequently, the measurement accuracy is lowest at high concentrations. The present study is advantageous in that only a single substance is detected, thus necessitating only the separation of water and CO₂ from sevoflurane via GC. In the measurement with the insufflated CO_2_ concentration, an additional peak is observed in addition to the sevoflurane, which may be correlated with the CO_2_ concentration. It should be noted that the measured concentrations refer only to the volume directly behind the anesthetic exhaust of the adsorber, which is the same sample position, where the SENSOfluran™ level indicator is located. Therefore, the sevoflurane concentrations will dilute quickly into the room depending on room size and air exchange rate resulting in a much lower workplace contamination (Fig. 2). We only carried out each experiment once, as the individual experiments build on each other and the results are therefore comprehensible.

## Conclusions

This study demonstrates that this anesthetic gas adsorber does not leak sevoflurane until the canister is almost saturated as evident from the built-in alert system. Anesthesiologists can safely change the canister once the yellow-light warning appears. However, the canister is not fully saturated at this point. The question arises as to whether an improvement in the sensor could contribute to an increase in the effectiveness and safety of the process, since delivery and return of adsorbers for recovery of volatile anesthetics lead to additional CO_2_ emissions.

## Data Availability

The datasets used and/or analyzed during the current study are available from corresponding author on reasonable request.

## Abbreviations

AGSS: Anesthetic gas scavenging systems
FGF: Fresh gas flow
GC: Gas chromatographic
GC-IMS: Ion mobility spectrometry with gas chromatographic
GWP: Global warming potenials
IMS: Ion mobility spectrometer
HFIP: Hexafluoroisopropranol
LCA: Lifecycle assessment
PFAS: Per-and polyfluoroalkyl substances
TDA: Trifluoroacetic acid
